# An infection prevention and control program established in the wake of the Ebola epidemic: Where are we, and how well are we doing?

**DOI:** 10.1017/ash.2023.328

**Published:** 2023-09-29

**Authors:** Bobson Fofanah, Christiana Conteh, Victoria Katawera, Ibrahim Franklyn Kamara

## Abstract

**Background:** Infection prevention and control (IPC) is a clinical and public health discipline based on a scientific approach and practical preventive and control measures. During the 2014–2016 West African Ebola outbreak, the high number of healthcare worker infections was attributed to inadequate IPC in Sierra Leone. This stimulated the establishment of national and subnational IPC programs. Since then, IPC has remained a priority to improve the health systems and strategic interventions during public health emergencies. Therefore, we conducted a detailed review to assess the status of the IPC programs. **Methods:** A descriptive analysis of the status of IPC programs in Sierra Leone was done using data from IPC assessments conducted in 2022 by the national IPC team, reviews of reports on program implementation, and experts’ objective opinions. **Results:** *Performance.* The national IPC assessment revealed strengths in 4 of 6 WHO IPC core components, with an overall score of 61% positioned at the ‘intermediate’ level of implementation. The best-performing component was ‘IPC guidelines’ (92%) with evidenced-based guidelines being developed and implemented over the years. Secondly, ‘Education and training’ (71%) made progress in basic and advanced IPC training, including the development of a preservice training curriculum. Also, ‘monitoring and audit and feedback’ (69%) and ‘IPC program’ (61%) met the basic requirements of an established Monitoring & Evaluation (M&E) system. Similar progress was made at the healthcare facility level, but with major gaps in ‘workload, staffing, bed-occupancy’ and ‘safe or built environment.’ *Sustainability efforts.* Evidence-based data on IPC have always been scarce due to a limited capacity to conduct IPC research. The Structured Operational Research and Training Initiative (SORT-IT) on antimicrobial resistance has helped promote evidence-informed decisions and build OR capacity that is relevant to improving program performance. In 2019, Sierra Leone instituted in-country production of alcohol-based handrub and liquid soap as a strategic intervention for providing hand hygiene products for use in healthcare facilities. This intervention was essential during the peak of the COVID-19 pandemic. Although most aspects of IPC implementation are government led with strong leadership support, stable funding and sustainability planning are yet to be achieved and will be crucial for long-term success. **Conclusions:** Most aspects of the IPC core components have been well implemented at the national level since the establishment of the IPC program. However, the program should continue improving the scope and quality of implementation and focus on the development of long-term plans to sustain existing gains and further improve on gap areas at the national level and especially the healthcare-facility level.

**Figure:**
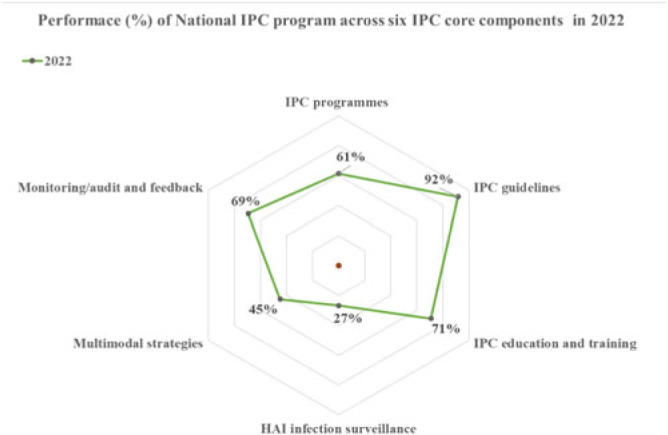

**Disclosures:** None

